# Exploring language use in apology strategies of tourists visiting Saudi Arabia

**DOI:** 10.1371/journal.pone.0319393

**Published:** 2025-02-25

**Authors:** Abdullah Al Fraidan, Abdulaziz A. Alhulaybi

**Affiliations:** 1 Department of English Language, College of Arts, King Faisal University, Al Ahsa, Saudi Arabia; 2 Department of Arabic Language, College of Arts, King Faisal University, Al Ahsa, Saudi Arabia; New York University, UNITED STATES OF AMERICA

## Abstract

This research explores the apology strategies employed by international tourists visiting Saudi Arabia, particularly in the context of the Kingdom’s rapid tourism expansion under Vision 2030. With the introduction of new tourism policies, the country has opened itself to a wide range of international visitors, increasing the likelihood of cultural missteps. The study investigates how tourists navigate Saudi Arabia’s conservative social norms, focusing on factors such as language use, cultural awareness, and the perceived severity of offenses. Data were collected through qualitative focus group interviews with international tourists, analyzing how apology strategies are influenced by social dynamics, gender norms, and power structures. The findings suggest that tourists who demonstrate cultural sensitivity, particularly by using Arabic, are more likely to have their apologies accepted. The study highlights the importance of cross-cultural competence in managing tourist-local interactions and provides insights for tourism operators and policymakers on improving the tourist experience in Saudi Arabia. The research contributes to a deeper understanding of cross-cultural communication in the context of a rapidly growing tourism industry.

## Introduction

Saudi Arabia, known for its rich Islamic heritage and deeply rooted cultural traditions, has long been a focal point for religious tourism. Historically, the Kingdom has attracted millions of Muslim pilgrims from around the world, particularly for the annual Hajj pilgrimage, one of the Five Pillars of Islam, and for Umrah, a non-mandatory pilgrimage that can be undertaken at any time of the year. Makkah and Madinah, the two holiest cities in Islam, have been the primary destinations for this influx of religious tourists, and Saudi Arabia has built much of its tourism infrastructure around catering to the needs of these pilgrims. However, for many years, the Kingdom limited tourism primarily to religious purposes, with only minimal engagement in broader leisure or cultural tourism sectors [1].

The launch of *Vision 2030* has marked a significant turning point in the Kingdom’s approach to tourism. In an effort to diversify its economy, reduce its reliance on oil revenues, and integrate more into the global economy, Saudi Arabia’s *Vision 2030* plan seeks to expand non-oil sectors such as entertainment, infrastructure [2,3], and, importantly, tourism. This ambitious vision aims to transform Saudi Arabia into a leading global tourism destination by promoting the country’s cultural, historical, and natural sites, in addition to its Islamic heritage. By 2030, the Kingdom aims to welcome over 100 million visitors annually, positioning itself as a cultural and tourism hub in the Middle East and beyond (Saudi Vision 2030, 2021).

To achieve these goals, the Saudi government has implemented several key initiatives. One of the most significant is the introduction of a new e-visa system in 2019, which allows tourists from over 49 countries to obtain visas online or upon arrival, simplifying the previously complex and restrictive process of visiting the Kingdom. This move has made Saudi Arabia more accessible to international travelers, including non-Muslim tourists who may not have been able to visit previously. In addition to the e-visa initiative, the government has invested heavily in developing state-of-the-art infrastructure, such as modern airports, hotels, transportation systems, and luxury resorts, to accommodate the anticipated influx of tourists [4].

These efforts are coupled with the promotion of cultural tourism beyond religious pilgrimages. Saudi Arabia is home to numerous UNESCO World Heritage sites, including the ancient Nabatean city of Al-Ula, the Diriyah Gate, and the historical coastal city of Jeddah. These locations, which highlight the rich history and cultural diversity of the Kingdom, are being marketed as key attractions for international tourists. Moreover, Saudi Arabia’s vast desert landscapes, mountainous regions, and Red Sea coastline offer opportunities for eco-tourism, adventure tourism, and luxury experiences, which are central to the government’s strategy for expanding its tourism sector [5]. These efforts to diversify the tourism sector align with the broader goals of *Vision 2030*, which include promoting cultural exchange, improving the country’s global image, and showcasing Saudi Arabia as a progressive and open society.

However, with this rapid influx of international tourists comes a new set of challenges. Saudi Arabia’s social and religious norms are deeply conservative compared to many of the countries from which tourists originate. As visitors from different cultural backgrounds engage with Saudi society, they may inadvertently violate these norms, leading to cultural misunderstandings and social missteps. For example, issues surrounding dress codes, gender segregation, and religious customs such as prayer times can often create situations where tourists unknowingly offend locals [6]. While Saudi Arabia is working to strike a balance between maintaining its cultural integrity and welcoming global tourists, the potential for such misunderstandings is high, particularly as the Kingdom’s tourist profile expands beyond its traditional base of religious pilgrims.

In this context, apology strategies become crucial for facilitating smooth cross-cultural interactions. When tourists violate local customs, whether knowingly or unknowingly, their ability to offer a culturally appropriate apology can make a significant difference in how their actions are perceived and how relationships with locals are maintained. Apologies, when given sincerely and in line with cultural expectations, can serve to restore social harmony, demonstrating the tourist’s respect for local values and a willingness to adapt their behavior accordingly [7].

This paper investigates the diverse apology strategies employed by tourists visiting Saudi Arabia, analyzing the key factors that influence these strategies. These factors include language use, cultural awareness, and the perceived severity of offenses, all of which play critical roles in determining how apologies are structured and received. For instance, tourists who make the effort to apologize in Arabic or who demonstrate a clear understanding of the cultural or religious significance of their offense are generally more likely to have their apologies accepted [8]. Conversely, tourists who rely solely on their native language or who fail to acknowledge the cultural weight of their actions may find that their apologies are less effective or even rejected.

In addition to examining these apology strategies, the paper also explores how *Vision 2030* is shaping the Kingdom’s tourism sector and the broader cultural implications for tourist-local interactions. As Saudi Arabia seeks to position itself as a global tourism destination, the role of cross-cultural communication becomes increasingly important. The government’s efforts to modernize its tourism infrastructure and promote cultural exchange are central to its success in achieving these goals. However, these efforts must be accompanied by an understanding of the cultural sensitivities that arise when international tourists engage with Saudi society. By analyzing how tourists navigate these sensitivities through apology strategies, this research contributes to a deeper understanding of the broader cultural dynamics at play in Saudi Arabia’s emerging tourism industry.

### Apology strategies and cultural factors

In Saudi Arabia, deeply ingrained cultural and religious norms shape the way social interactions unfold, making it essential for tourists to respect and understand these traditions. The Kingdom’s collectivist culture places great emphasis on maintaining social harmony, respect for authority, and adherence to religious customs. This means that when tourists breach these norms, an apology is often required to restore social order and demonstrate respect for Saudi values [4].

Apologies in Saudi Arabia must be tailored to the context in which the offense occurs. For example, violating the local dress code, particularly for women, is considered a serious transgression. Saudi Arabia enforces conservative dress standards, particularly in public spaces, where modesty is not only a cultural preference but also a legal requirement. Tourists who unknowingly wear inappropriate clothing must issue apologies that acknowledge the transgression, express regret, and indicate a willingness to adhere to local customs moving forward.

Similarly, religious customs, such as the observance of prayer times, require a high level of respect from tourists. Prayer is a central aspect of daily life in Saudi Arabia, and tourists who interrupt or disregard prayer times may be seen as disrespectful. In such cases, a simple verbal apology may not suffice. The tourist must demonstrate an understanding of the religious significance of their transgression, often through both verbal and non-verbal cues, such as stepping aside during prayer or adopting a respectful posture.

Saudi society places a strong emphasis on honor and maintaining face in public interactions [9]. Apologies are not only directed at individuals but can also serve to restore social harmony within the broader group. For instance, public offenses—such as inappropriate behavior in a mosque or marketplace—require apologies that address both the offended individual and the collective group that witnessed the offense. Tourists who fail to recognize the collective dimension of Saudi society may find that their apologies fall short of expectations.

### Language use in apologies

Language plays a vital role in shaping the effectiveness of apologies, particularly in a multicultural context like Saudi Arabia, where Arabic is the official language and carries deep cultural significance. Apologies delivered in Arabic are often seen as more respectful and sincere, especially when the offense involves religious or cultural norms. As [8] note, language choice in cross-cultural communication can significantly impact how apologies are perceived, with apologies offered in the local language often viewed as more genuine.

Many tourists visiting Saudi Arabia, however, may not speak Arabic fluently and may instead rely on English, which is widely used in urban areas and the tourism sector. While English-language apologies are generally understood, they may lack the cultural resonance of an apology offered in Arabic. For instance, a tourist who apologizes in English after accidentally entering a mosque without appropriate attire may be understood, but the apology may not carry the same weight as if it were delivered in Arabic. This distinction is particularly important in cases involving religious or cultural transgressions, where the use of Arabic demonstrates a deeper level of cultural awareness.

Vision 2030 encourages greater cultural exchange and language learning, recognizing that linguistic competence is key to fostering positive interactions between tourists and locals. As part of these efforts, the Saudi government has implemented programs to teach tourists basic Arabic phrases, including expressions of apology, to help bridge the cultural gap. Tourists who make an effort to use Arabic in their apologies, even if only in simple phrases like “Aasif” (I’m sorry), are more likely to have their apologies accepted and appreciated by locals [8].

### Factors influencing apology strategies

Several factors influence the apology strategies employed by tourists in Saudi Arabia, including the perceived severity of the offense, the relationship between the individuals involved, and the power dynamics at play.

#### Severity of the offense.

The perceived severity of an offense plays a major role in shaping the formality of the apology. In Saudi Arabia, offenses that violate religious customs, such as interrupting prayer or dressing immodestly, are considered serious and require more formal apologies. In such situations, tourists must demonstrate a clear understanding of the cultural importance of their transgression and offer apologies that go beyond mere verbal expressions of regret. Non-verbal cues, such as bowing the head or avoiding direct eye contact, can enhance the sincerity of the apology and demonstrate respect for local customs [10].

#### Social distance and power dynamics.

The formality and tone of an apology are also shaped by the relationship between the apologizer and the offended party. In Saudi Arabia, where respect for authority and social hierarchy is emphasized, apologies directed at authority figures, such as religious leaders or government officials, must be formal and deferential. Tourists who fail to recognize the power dynamics at play may offer apologies that are perceived as disrespectful, exacerbating the offense rather than resolving it.

For instance, a tourist who apologizes casually to a religious leader after violating a mosque’s dress code may deepen the offense if the apology does not reflect the leader’s social status. In contrast, apologies between peers or in informal settings may be more direct, although even in these situations, tourists must remain mindful of Saudi Arabia’s cultural norms regarding respect and honor [11].

#### Gender norms.

Gender plays a significant role in shaping apology strategies in Saudi Arabia, where strict gender segregation governs many aspects of public life. Female tourists, in particular, may face challenges in navigating these norms, especially when they inadvertently breach them. Apologies in such cases must not only acknowledge the specific offense but also demonstrate cultural awareness of Saudi Arabia’s gender norms.

For example, a female tourist who accidentally brushes against a man in a crowded marketplace may need to offer a culturally appropriate apology that reflects her understanding of the social transgression. By demonstrating respect for local customs and offering a sincere apology, tourists can mitigate the negative impact of such offenses and foster positive interactions with locals [12].

## Literature review

The literature on apology strategies in cross-cultural settings provides valuable insights into how tourists navigate social interactions in unfamiliar cultural contexts [7]. seminal work on speech acts highlights the role of context in shaping apology strategies, showing that the content and form of an apology are influenced by the cultural norms of the society in which it takes place. In collectivist cultures like Saudi Arabia, where public behavior is closely tied to social harmony, apologies must often address both the individual and the broader group to restore balance [11]. Research on cross-cultural communication underscores the importance of language in apology strategies, particularly in multilingual settings. They argue that apologies offered in the local language are often perceived as more sincere and respectful, particularly in cases involving cultural transgressions. This is especially relevant in Saudi Arabia, where the use of Arabic in apologies demonstrates cultural sensitivity and a willingness to engage with local norms [8]. Emphasize the importance of cultural competence in navigating cross-cultural interactions. Their research suggests that individuals who possess a higher degree of cultural awareness are better equipped to adapt their apology strategies to the cultural context, resulting in more effective communication. In Saudi Arabia, where cultural norms are often unfamiliar to international tourists, this cultural competence is crucial for ensuring that apologies are understood and accepted by locals [13].

Additional research by [14] on politeness theory also supports the idea that apology strategies vary depending on social context and the nature of the offense. In societies like Saudi Arabia, where respect for authority and adherence to religious customs are paramount, tourists must adopt more formal and respectful apology strategies, particularly when interacting with figures of authority.

Prior studies on tourist motivation and behavior in Saudi Arabia and the wider Arab Muslim culture are limited [6]. However, several studies have explored apology strategies in Arabic and Islamic contexts, offering valuable insights into how cultural and religious norms shape communicative behaviors. For instance, [15] examined how non-native speakers navigate Arabic apology strategies, highlighting the influence of linguistic proficiency and cultural awareness on their effectiveness. Similarly, [16] conducted a cross-cultural study comparing apologies in Arabic and English, emphasizing the role of politeness and face-saving strategies in shaping apology behaviors. Explored the intersection of gender and apology strategies in Arabic-speaking societies, providing a nuanced understanding of how0 cultural and social variables influence speech acts in Muslim contexts [17]. Identified significant variations in apology strategies based on social variables such as age, gender, and the severity of the offense in Jordanian Arabic and English. These findings underscore the complexity of apology behaviors in collectivist cultures [18]. Explored the pragmatics of apologies in Iraqi Arabic and English, identifying how cultural and linguistic factors influence the choice of strategies [19]. Investigated apologies in Saudi and Australian contexts, providing cross-cultural insights into how gender and cultural background affect communication. These studies collectively highlight the interplay between language, culture, and social expectations in shaping apologies [20,21].

These studies underscore the complexity of apologies in the Muslim world, particularly within collectivist cultures where maintaining social harmony and respect is paramount [22]. However, existing research largely focuses on local interactions or comparisons between Arabic speakers and non-Arabic speakers. Few studies have examined how international tourists navigate apology strategies when engaging with locals in Saudi Arabia, particularly in the context of the Kingdom’s growing tourism sector under Vision 2030. This study aims to fill this gap by exploring the diverse apology strategies employed by international tourists and analyzing how cultural, linguistic, and social factors influence these behaviors.

## Methodology

This study employed a qualitative research methodology, using in-depth focus group interviews to explore the apology strategies used by international tourists visiting Saudi Arabia. While focus groups provide rich, descriptive data on cultural nuances and social interactions, they inherently limit the generalizability of findings due to the small sample size and specific participant demographics. The focus group method was chosen for its ability to elicit detailed, context-specific insights into participants’ experiences, which are particularly valuable for exploring the complex interplay of language, culture, and social norms. Future research could complement these findings with quantitative methods, such as large-scale surveys or experiments, to enhance the generalizability of results.

Qualitative research is well-suited for studies that aim to uncover rich, in-depth insights into participants’ lived experiences, particularly in culturally nuanced contexts [23] By focusing on how tourists navigate social interactions in the Saudi context, particularly in situations that require apologies, this study allows for a detailed understanding of the complexities of cross-cultural communication.

Focus group interviews were chosen because they provide a dynamic setting in which participants can share their experiences, challenge each other’s views, and collectively reflect on the cultural differences and communication challenges they faced. The interactive nature of focus groups can generate deeper insights than one-on-one interviews, as participants often build upon each other’s responses, leading to richer data [24]. The discussions also facilitated the exploration of multiple perspectives on how tourists experience social norms in Saudi Arabia, particularly in situations where an apology was required due to a perceived cultural transgression.

The focus groups were semi-structured to balance guiding the conversation while allowing for flexibility in the responses. Open-ended questions were used to encourage participants to reflect on their specific experiences, the language used in these interactions, and the factors that influenced their apology strategies. This flexible structure ensured that while key topics were covered, participants also had the opportunity to bring up issues and experiences that may not have been anticipated by the researchers, offering richer, more spontaneous data [23].

The qualitative nature of the focus group discussions also allowed participants to reflect on the role of cultural norms in shaping their behavior. Many of the participants had never before encountered a social environment like Saudi Arabia’s, which is characterized by strict adherence to Islamic values and traditions. This unfamiliarity often led to unintentional social transgressions, such as violations of dress codes or misunderstandings related to religious practices like daily prayers. The focus group setting encouraged participants to share how they handled these situations, particularly in terms of offering apologies, which were often shaped by their awareness—or lack thereof—of Saudi customs.

The qualitative approach was essential for capturing the emotions, reflections, and subtle shifts in behavior that tourists experienced as they became more culturally attuned to their environment. The richness of the data gathered through focus groups provided an opportunity to explore how tourists responded to social feedback, how they learned to navigate local norms, and how they adapted their apology strategies based on this feedback. The methodology allowed for a more nuanced analysis of apology strategies than quantitative data alone would have provided.

### Ethical statement

This study adhered to the ethical guidelines set forth by King Faisal University Ethics Committee under the approval no. 717.

### Participants

A total of 20 international tourists participated in the study. The participants were recruited at key tourist destinations in Riyadh, Jeddah, and Mecca, areas that have experienced significant tourism infrastructure development as part of Saudi Arabia’s Vision 2030 initiative (Saudi Vision 2030, 2021). The recruitment period spanned from 01/03/2024 to 22/03/2024, ensuring that participants were selected during the peak tourism season, which allowed for a rich diversity of experiences to be captured.

Participants were selected based on specific inclusion criteria to ensure they could meaningfully contribute to the study. To participate, individuals had to be international tourists visiting Saudi Arabia, aged 21 or older, to ensure they could provide reflective and mature insights into their experiences. Additionally, participants needed to be willing and able to engage in English-language focus group discussions. While the discussions were conducted in English, any non-English phrases used by participants during their apologies were analyzed as part of the data. These criteria were designed to ensure that the participants could actively engage in the discussions and offer diverse perspectives on apology strategies across different cultural contexts

The participant pool included a balanced mix of leisure and religious tourists, drawn from North American, European, Asian, and Middle Eastern countries. This cultural diversity was essential for understanding how individuals from varying cultural contexts navigate social interactions in Saudi Arabia. The group consisted of 12 women and 8 men, with ages ranging from 21 to 65 years, representing a wide spectrum of perspectives on gender and generational influences in cross-cultural interactions.

Among the participants, some were visiting Saudi Arabia for the first time, while others were repeat visitors who had prior familiarity with the Kingdom’s cultural norms. This mix allowed for a comparison between first-time visitors, who often relied on their assumptions or prior knowledge about Middle Eastern culture, and repeat visitors, who had adapted their behaviors based on past experiences. Moreover, the study highlighted the contrast in apology strategies between tourists from individualistic cultures (e.g., North America and parts of Europe) and those from collectivist societies (e.g., Asia and the Middle East) [22].

Some participants were visiting Saudi Arabia for the first time, while others had previously traveled to the Kingdom. This mix allowed the study to compare how first-time visitors’ apology strategies differed from those of repeat visitors, who may have had more familiarity with Saudi norms and thus could reflect on how their approaches evolved over time. Moreover, the diversity in cultural backgrounds highlighted the differences in how tourists from individualistic cultures (such as those from North America and parts of Europe) navigated the collectivist cultural context of Saudi Arabia compared to those from more collectivist societies (such as parts of Asia and the Middle East) [22].

The range of participant demographics provided a robust dataset for understanding how cultural background, gender, and familiarity with the Kingdom influenced apology strategies. For example, women from more liberal Western cultures encountered unique challenges related to Saudi Arabia’s strict gender segregation policies, and their experiences of having to apologize for gender-related transgressions provided critical insights into the intersection of gender and cultural norms in the Kingdom.

### Data collection

This study adhered to the ethical guidelines set forth by King Faisal University Ethics Committee. All participants provided written informed consent prior to their involvement in the research. During the consent process, participants were informed about the study’s purpose, the voluntary nature of their participation, their right to withdraw at any time, and the confidentiality of their responses.

Data were collected through semi-structured focus group interviews, each lasting between 60 and 90 minutes. The semi-structured format was ideal for encouraging open dialogue while also ensuring that key areas of inquiry were covered consistently across all focus group [24]. The flexibility of this approach allowed participants to delve into specific topics in depth, while the interview guide ensured that discussions addressed important themes such as instances where apologies were necessary, the language used during apologies, and how cultural dynamics shaped these interactions.

The focus groups were conducted in English, as this was the most common language among the participants. However, participants were encouraged to reflect on their use of Arabic or their native languages when offering apologies in Saudi Arabia. This bilingual reflection was particularly important for understanding how language choice influenced the effectiveness of the apology and how it was received by local Saudi nationals [8]. For example, participants discussed situations where using Arabic phrases like “Aasif” (I’m sorry) helped convey sincerity, even when they were not fluent in the language, highlighting the cultural significance of language in apology strategies.

The data collection process also captured the complexities of how tourists navigated cultural differences and communication challenges during their stay in Saudi Arabia. Tourists frequently discussed the role of non-verbal communication in their apology strategies, such as body language and gestures of respect, which are particularly important in a conservative cultural context like Saudi Arabia’s. Participants recounted instances where they inadvertently violated local norms, such as interrupting prayer or dressing inappropriately, and reflected on how they adapted their apologies based on local feedback.

The focus group discussions were recorded and later transcribed verbatim to ensure accuracy in capturing the nuances of the conversations. Thematic analysis was then employed to identify recurring patterns and themes related to apology strategies [25]. This approach allowed the researchers to systematically explore the data and draw connections between the language used in apologies, the severity of the offense, and how social dynamics—including power relations, gender, and authority—shaped the participants’ responses.

The thematic analysis focused on three key areas: language use, the perceived severity of the offense, and the influence of cultural and social dynamics. By categorizing and coding the data in this way, the researchers were able to identify how different factors influenced apology strategies and how participants adapted their approaches in response to the cultural context. For example, the analysis revealed that participants who were more familiar with Saudi customs often employed more nuanced apology strategies, reflecting their understanding of the cultural weight of their transgressions. In contrast, first-time visitors often relied on more direct, sometimes insufficient, apologies, which were less effective in restoring social harmony.

To ensure the reliability of the analysis, two independent raters coded the data separately. The raters achieved a high inter-rater reliability score (r =  0.92), indicating a strong level of agreement. Discrepancies in coding were resolved through discussion and consensus, ensuring that the final themes accurately represented the participants’ experiences. This rigorous analytical process enhanced the validity of the findings and provided a robust framework for interpreting the data.

In addition to language use, the analysis examined the role of power dynamics in apology scenarios. In Saudi Arabia, where social hierarchy and respect for authority figures such as religious leaders and law enforcement officials are critical, participants described how they adapted their apologies to reflect these power relations. For instance, some tourists recounted offering more formal and deferential apologies when dealing with figures of authority, recognizing that casual apologies could be perceived as disrespectful in such contexts [22].

By examining these elements, the data collection process provided valuable insights into how tourists navigate apology strategies in Saudi Arabia, offering practical implications for improving cross-cultural communication and enhancing the tourist experience in the Kingdom.

## Results

The findings from the focus groups revealed several key insights. Tourists often found themselves in situations where they needed to offer apologies, such as inadvertently violating dress codes by wearing clothing deemed inappropriate or immodest, speaking too loudly in public spaces where quietness is expected, or engaging in other behaviors that were deemed inappropriate or disrespectful within the conservative Saudi cultural context. These situations highlighted the cultural differences and sensitivities that tourists had to navigate during their travels in Saudi Arabia.

The findings from the focus groups revealed several key insights, which are organized under the following themes: cultural sensitivity, language use in apologies, and gender and power dynamics. Each theme is supported by representative participant quotes to illustrate the nuanced experiences of international tourists in Saudi Arabia.

### Cultural sensitivity and apology strategies

One of the most significant findings was the importance of cultural sensitivity in shaping apology strategies. Tourists who demonstrated an understanding of Saudi cultural norms and attempted to adapt their behavior were more likely to have their apologies accepted. For example, participants who made an effort to use Arabic phrases, such as “Aasif” or “Shukran,” reported more positive interactions with locals, particularly in situations where the offense involved a cultural or religious transgression. One participant, a female tourist from the United Kingdom, shared an experience where she unintentionally wore clothing that did not adhere to local modesty standards. When a local woman pointed out the issue, she quickly apologized in Arabic and adjusted her outfit. The tourist noted that her use of Arabic, even though it was limited, seemed to be appreciated by the local woman, who responded with kindness and understanding. This example illustrates how even a small effort to engage with the local language can enhance the effectiveness of an apology in a cross-cultural setting.

Tourists frequently encountered situations where they unknowingly breached cultural norms, such as dress codes, prayer etiquette, or public behavior expectations. Their apology strategies reflected a growing awareness of cultural sensitivity over time. This aligns with [7] observation that apologies in collectivist cultures serve as face-saving acts to restore social harmony.

A female tourist from the United Kingdom shared her experience:

*“When the woman pointed out my dress was inappropriate, I immediately said ‘Aasif’ [I’m sorry] and adjusted it. Her smile told me she appreciated my effort to respect her culture.”*

This example illustrates how tourists adapted their behavior to align with local expectations, which [18] highlights as critical in cross-cultural apology strategies. The use of culturally embedded language demonstrates the significance of linguistic effort in fostering understanding and acceptance.

### Language proficiency and effectiveness

Language emerged as a critical factor in how apologies were received by locals. Participants who used Arabic, even in a limited capacity, reported more successful interactions than those who relied solely on English or their native language. This finding aligns with previous research suggesting that apologies offered in the local language are perceived as more sincere and respectful [8]. However, not all participants felt confident using Arabic, particularly in situations where the offense involved complex religious or social norms.

For instance, a male tourist from Canada recounted an experience where he unintentionally interrupted a prayer service at a mosque. Although he apologized in English, he felt that the apology was not fully understood or accepted by the worshippers. Reflecting on the experience, he noted that using Arabic might have made the apology more effective, as it would have shown a greater respect for the cultural and religious context of the situation.

The choice of language played a crucial role in how apologies were received by locals. Tourists who attempted to use Arabic phrases, even minimally, reported more positive responses than those who relied solely on English.

- A French tourist noted:

*“I struggled with Arabic, but even saying ‘Shukran’ [thank you] or ‘Aasif’ helped. People appreciated the effort, and it softened the situation when I made a mistake.”*

- An American participant stated:

*“I apologized in English, but it didn’t seem to have the same impact. When I added an Arabic word, I noticed a change in their reaction—they seemed more understanding.”*

These accounts suggest that even limited attempts to engage in the local language enhance the perceived sincerity of apologies, reflecting the cultural significance of language in Saudi Arabia.

### Gender and apology strategies

Gender was another important factor influencing apology strategies. Female participants, in particular, faced unique challenges related to Saudi Arabia’s strict gender segregation rules. Several female participants reported situations where they inadvertently violated these norms, such as by making physical contact with a man in a public space. In these cases, female tourists who offered apologies that demonstrated cultural sensitivity and a willingness to adhere to local customs were more likely to receive forgiveness from locals.

One female participant from France described an incident in which she accidentally brushed past a man in a busy market. She immediately apologized in Arabic, acknowledging the cultural norm she had violated. The man accepted her apology and even helped her navigate the crowded space. This example underscores the importance of gender-specific apology strategies in Saudi Arabia, where interactions between men and women are governed by strict cultural and religious protocols.

Gender norms significantly influenced the nature and effectiveness of apology strategies. Female participants often expressed greater concern about adhering to local norms and adapting their behavior to avoid potential offenses.

- A female participant from France reflected:

*“As a woman, I felt more pressure to be respectful and avoid offending anyone. When I apologized, I made sure to use a softer tone and smile—it seemed to make a difference.”*

- In contrast, a male tourist from Australia observed:

*“I didn’t feel the same need to apologize as often. But when I did, I kept it short and direct—it worked fine in most cases.”*

These differences underscore how gendered expectations shape cross-cultural interactions and apology strategies in Saudi Arabia’s conservative context.

### The influence of power dynamics

The findings also highlighted the influence of power dynamics on apology strategies. Participants who interacted with authority figures, such as religious leaders or law enforcement officers, often adopted more formal and deferential apology strategies. This reflects the hierarchical nature of Saudi society, where deference to authority is an important cultural value. Participants who failed to recognize these power dynamics and offered more casual apologies were often met with disapproval, deepening the offense rather than resolving it.

One participant, an American tourist, shared an experience where he accidentally took photographs in a restricted area near a mosque. When approached by a security officer, he offered a casual apology in English, which was not well received. Upon reflection, the tourist realized that a more formal apology, possibly in Arabic, would have been more appropriate given the context and the authority of the security officer.

Apologies directed toward authority figures, such as police officers or religious leaders, were notably more formal and deferential, reflecting the hierarchical nature of Saudi society.

- An American tourist recounted:

*“I accidentally walked into a mosque without removing my shoes. When I realized my mistake, I apologized profusely and lowered my gaze. The imam accepted my apology but reminded me to be more careful.”*

- A British participant shared:

*“I bumped into a guard at a historical site and immediately apologized using Arabic. He appreciated the gesture, and it helped avoid further tension.”*

These examples illustrate how power dynamics influence apology strategies, requiring tourists to adopt more respectful and formal approaches in interactions with authority figures.

## Discussion

The findings of this study underscore the importance of language and cultural sensitivity in shaping apology strategies, particularly in a collectivist and hierarchical society like Saudi Arabia. Tourists who attempted to use Arabic phrases, even minimally, experienced more favorable responses from locals. This supports [7] theory that speech acts are culturally embedded and align with politeness norms. Similarly, [18] emphasizes that using culturally appropriate language enhances the perceived sincerity of apologies in Arabic-speaking contexts.

Gender norms also influenced the nature of apology strategies. Female participants often employed more elaborate and conciliatory approaches, reflecting societal expectations of politeness and deference [17]. For instance, a female French tourist shared:

*“As a woman, I felt the need to smile and use a softer tone in my apologies. It seemed to make people more receptive.”*

In contrast, male tourists frequently adopted more direct strategies, which were generally well-received in interactions with other men. These findings highlight how apology strategies are mediated by both gender and cultural expectations, reinforcing the significance of adapting to local norms for successful cross-cultural communication

The results also align with [22] framework on collectivist cultures, which prioritize group cohesion over individual preferences. In Saudi Arabia, tourists who demonstrated an understanding of local norms and values were more successful in mitigating offenses, highlighting the importance of deference and humility in cross-cultural interactions. This finding underscores the influence of collectivist values, where apologies serve not only to repair relationships but also to uphold societal expectations of respect and harmony.

In contrast to studies on individualist cultures, where apologies may emphasize personal responsibility and directness, the strategies employed in this study often reflected a more indirect and relational approach. For example, tourists frequently combined verbal apologies with non-verbal gestures, such as lowering their gaze or adopting a deferential tone, which mirrors [11] politeness theory. These behaviors suggest a sensitivity to the hierarchical and gendered dynamics inherent in Saudi society.

Gender norms also played a significant role, with female participants expressing greater concern about the appropriateness of their behavior and the effectiveness of their apologies. This aligns with [17] findings on gendered differences in apology strategies in Arabic-speaking cultures, where women often adopt more elaborate or conciliatory approaches to mitigate perceived offenses. These patterns underscore the intersection of cultural and gendered expectations in shaping apology strategies in Saudi Arabia.

Power dynamics further influenced the success of apologies, particularly in interactions with authority figures. Participants described adapting their language and tone to reflect the hierarchical nature of Saudi society, echoing [7] assertion that social status significantly affects the formality and content of speech acts. This finding highlights the necessity of understanding and navigating power relations in cross-cultural interactions.”

### The role of language in cross-cultural apologies

One of the key contributions of this study is its focus on the role of language in shaping apology strategies. The data suggest that tourists who make an effort to use Arabic when offering apologies are more likely to foster positive interactions with locals. This finding is consistent with previous research on the importance of language in cross-cultural communication [8]. In the context of Vision 2030, which promotes cultural exchange and the integration of tourists into Saudi society, the use of Arabic serves as a powerful tool for building rapport and demonstrating respect for local norms.

However, the effectiveness of language use in apologies also depends on the tourist’s level of proficiency and understanding of cultural norms. Tourists who use Arabic without fully grasping its cultural nuances risk offering apologies that are perceived as insincere or inappropriate. This highlights the need for greater cultural and linguistic competence among international tourists, particularly those visiting countries with strong cultural and religious traditions.

### The role of power dynamics and gender norms in apology success

Power dynamics and gender norms emerged as significant factors influencing the success of apology strategies in Saudi Arabia. Participants reported adapting their apologies to reflect the hierarchical nature of Saudi society, where respect for authority figures and adherence to cultural protocols are paramount. For example, apologies directed toward religious leaders, law enforcement officers, or business owners often required a higher level of formality and deference.

One participant recounted an interaction with a religious leader:

*“I accidentally entered a mosque without removing my shoes. When I realized my mistake, I apologized profusely, using a humble tone and lowering my gaze. The imam accepted my apology but reminded me to be more mindful. I think the way I showed respect helped him forgive me.”*

This observation aligns with [7] findings on speech acts in hierarchical cultures, where formal and deferential language is critical in interactions with authority figures. The success of apologies in these scenarios often depended on the offender’s ability to acknowledge the power imbalance and demonstrate genuine regret.

Gender norms also played a critical role in shaping apology strategies and their reception. Female participants expressed greater concern about conforming to local expectations and often employed more elaborate or conciliatory apology strategies. For instance, women frequently paired verbal apologies with non-verbal gestures such as smiling or adopting a softer tone to convey sincerity.

A female participant from France shared her experience:

*“I felt I had to apologize more carefully to avoid offending anyone. I used a polite tone and smiled—it seemed to make a difference.”*

In contrast, male participants often adopted more straightforward and less expressive apology strategies, which were generally well-received in interactions with other men. This aligns with [17] findings that women tend to employ more mitigating strategies in apologies due to societal expectations of femininity and politeness.

Moreover, the success of apologies in Saudi Arabia’s gender-segregated society was influenced by the gender of both the offender and the recipient. For example, apologies from male tourists to female locals often required a heightened level of respect and cultural sensitivity to avoid misunderstandings. Similarly, female tourists interacting with male authority figures reported feeling a need to overcompensate in their apologies to ensure their actions were not perceived as disrespectful.

These findings underscore the interplay between power dynamics and gender norms in shaping cross-cultural interactions and apology strategies. While hierarchical and gendered expectations may pose challenges for international tourists, understanding and adapting to these norms can significantly enhance the effectiveness of their apologies and foster positive interactions with locals.

### Analyzing local perceptions and responses to apologies

“The findings of this study suggest that locals’ responses to apologies are influenced by a combination of linguistic effort, cultural sensitivity, and the perceived sincerity of the tourist’s actions. While locals were generally forgiving when tourists acknowledged their mistakes, their responses varied in degree. For example, participants reported that using Arabic phrases, even minimally, often elicited more favorable reactions, reflecting the cultural significance of language as a marker of respect and effort. This aligns with [7] observation that speech acts are context-dependent and culturally embedded.

Moreover, the type of offense appeared to shape locals’ reactions. Minor offenses, such as dress code violations, were often met with understanding if the tourist quickly adapted their behavior. However, more significant cultural breaches, such as entering a mosque improperly, required formal and deferential apologies to restore social harmony. These findings highlight the importance of tourists demonstrating not only regret but also a willingness to learn and adapt to local norms.

Future research should include local participants to gain a direct understanding of their perceptions, enabling a more comprehensive analysis of cross-cultural interactions

### Theoritcal implications

The findings of this study align closely with established frameworks in intercultural pragmatics and politeness theory, offering insights into how tourists navigate apology strategies in a culturally complex context like Saudi Arabia. Framework on speech acts highlights that apologies are context-dependent, shaped by the cultural and linguistic norms of the interlocutors [7]. In Saudi Arabia, a collectivist society where social harmony is paramount, apologies are often designed to repair relationships and uphold mutual respect. Tourists who adapted their apologies to reflect these cultural priorities—such as by using deferential language or incorporating Arabic phrases—experienced greater success in mitigating offenses.

For example, a Canadian tourist recounted:

*“I apologized to a shopkeeper after walking in during prayer time. I added an Arabic phrase to show respect. His reaction shifted immediately—he seemed more understanding.”*

This observation reflects the importance of linguistic effort as a politeness strategy, consistent with [11] theory that face-saving acts are crucial in maintaining social harmony, particularly in hierarchical or collectivist cultures.

Politeness theory further explains the variations in apology strategies observed among tourists from individualist and collectivist cultures. Tourists from individualist societies, such as North America and Europe, initially relied on direct apologies, reflecting their cultural preference for personal accountability. However, those who quickly adapted to the indirect, relationship-oriented norms of Saudi society achieved better outcomes. This aligns with [11] distinction between positive politeness strategies (e.g., efforts to build rapport) and negative politeness strategies (e.g., showing deference).

Gendered and hierarchical dynamics in Saudi Arabia also resonate with these theoretical frameworks. Female tourists often employed more elaborate apologies, incorporating both verbal and non-verbal politeness strategies, to align with societal expectations of femininity and respect. Similarly, interactions with authority figures required heightened levels of deference, as seen in one participant’s account of apologizing to a religious leader:

*“I used a humble tone and lowered my gaze—it seemed necessary to show respect in that situation.”*

This behavior reflects [11] emphasis on negative politeness as a means of addressing power imbalances and avoiding further imposition.

Overall, the findings support the applicability of intercultural pragmatics and politeness theory in understanding how apology strategies are employed and received in cross-cultural contexts. By situating these strategies within broader theoretical frameworks, this study contributes to the growing body of research on speech acts and intercultural communication.

### Limitations

A key limitation of this study is the reliance on focus group data, which, while offering depth and context, may not fully capture the diversity of tourist experiences in Saudi Arabia. The small sample size and specific demographic composition of participants restrict the extent to which findings can be generalized. Future studies should aim to address this limitation by incorporating larger, more diverse samples or employing mixed methods approaches to triangulate findings. For instance, quantitative surveys could provide a broader understanding of apology strategies across different tourist populations, while experiments could test the efficacy of specific strategies in controlled settings.

This study specifically focused on exploring the apology strategies employed by international tourists visiting Saudi Arabia. While the inclusion of local Muslim men and women as participants could provide valuable insights into how apologies are perceived and responded to within the local cultural context, this was beyond the scope of the current study. Future research could address this gap by incorporating local perspectives, enabling a deeper understanding of the dynamics between tourists and locals, including the role of gender and cultural background in shaping apology interactions.

This study provides valuable insights into the apology strategies employed by international tourists visiting Saudi Arabia; however, it is not without limitations. The study relied on focus group data, which, while providing rich, descriptive insights, inherently limits the generalizability of the findings due to the small sample size and participant demographics. Additionally, the focus groups were conducted with tourists visiting urban settings, such as Riyadh, Jeddah, and Mecca, which are hubs of tourism infrastructure and cultural activity. As a result, the study does not capture the dynamics of cross-cultural interactions in rural or less-developed regions of Saudi Arabia, where norms and expectations may differ significantly.

Future research should address this limitation by including rural settings to explore whether apology strategies and cultural exchanges vary across different geographical and socio-economic contexts within the Kingdom. This would provide a more holistic understanding of how cultural settings influence cross-cultural interactions and apology strategies.

## Conclusion

In conclusion, this study offers a comprehensive analysis of apology strategies employed by international tourists visiting Saudi Arabia, shedding light on the multifaceted nature of cross-cultural communication. By examining the linguistic, cultural, and social dimensions of apologies, the research emphasizes the critical role of cultural adaptation and contextual awareness in fostering successful interactions in a culturally conservative and hierarchical society. The findings underscore that tourists who demonstrate cultural sensitivity, particularly through visible efforts to engage with local customs and language, often experience more positive responses to their apologies. The use of Arabic phrases, even minimally, emerged as a significant factor in bridging cultural gaps, reflecting the value placed on linguistic effort and respect within the Saudi context.

Furthermore, the study delves into the intricate influence of social variables such as gender and power dynamics on apology strategies. Female tourists were found to employ more elaborate and conciliatory approaches, aligning with societal expectations of politeness and relational sensitivity, while male tourists often favored direct and less expressive strategies. These patterns highlight the nuanced ways in which societal norms shape communication behaviors, offering valuable insights into the intersection of culture, gender, and language.

The role of power dynamics further adds to the complexity of apology strategies, particularly in interactions with authority figures. Tourists navigating hierarchical relationships, such as those with religious leaders or local officials, often adopted highly formal and deferential strategies to convey respect and mitigate offenses. This underscores the importance of understanding and adhering to local power structures in ensuring effective communication.

Overall, this study contributes to the broader discourse on intercultural pragmatics and cross-cultural communication by highlighting the unique challenges and strategies involved in navigating apology scenarios in Saudi Arabia. By focusing on the interplay of language, culture, and social norms, the research enriches our understanding of how speech acts function as tools for maintaining social harmony and fostering mutual respect in diverse cultural settings.

### Implications for tourism and policy

The findings of this study have several important implications for tourists, tourism operators, and policymakers in Saudi Arabia. First, the study highlights the need for greater cultural awareness and sensitivity among international tourists visiting the Kingdom. Tourists who are unfamiliar with local customs, particularly those related to dress codes, gender norms, and religious practices, may find themselves needing to apologize for unintentional offenses. To mitigate these challenges, tourism operators should consider providing cultural orientation programs that educate tourists about Saudi customs and norms, including appropriate apology strategies.

Second, the study underscores the importance of language proficiency in cross-cultural communication. Tourists who make an effort to use Arabic when offering apologies are more likely to have their apologies accepted by locals, particularly in situations involving religious or cultural offenses. Tourism operators and language schools could offer basic Arabic language courses to tourists, focusing on key phrases and cultural contexts that are likely to arise during their stay.

Third, the findings suggest that policymakers in Saudi Arabia should consider implementing more culturally sensitive tourism policies. While Saudi Arabia’s rich cultural and religious heritage offers unique opportunities for international tourists, it also presents challenges for those unfamiliar with local norms. Policymakers could develop guidelines or informational materials that provide tourists with practical advice on how to navigate social interactions, including when and how to offer apologies.

Finally, future research should explore how apology strategies differ across other regions of the Arab world, as well as in other culturally rich tourism destinations. Comparative studies could provide further insights into the role of language, culture, and power dynamics in shaping apology behavior, helping to inform more effective tourism strategies in diverse cultural contexts.
